# A systematic review of episodic volunteering in public health and other contexts

**DOI:** 10.1186/1471-2458-14-992

**Published:** 2014-09-24

**Authors:** Melissa K Hyde, Jeff Dunn, Paul A Scuffham, Suzanne K Chambers

**Affiliations:** Griffith Health Institute, Griffith University, Gold Coast Campus, Kragujevac, QLD 4222 Australia; Cancer Council Queensland, Spring Hill, QLD Australia; School of Social Science, University of Queensland, St Lucia, QLD Australia; Centre for Applied Health Economics, School of Medicine, Griffith University, Meadowbrook, QLD Australia; Prostate Cancer Foundation of Australia, St Leonards, NSW Australia; Health and Wellness Institute, Edith Cowan University, Perth, WA Australia; Centre for Clinical Research, University of Queensland, Herston, QLD Australia

**Keywords:** Episodic volunteering, Systematic review, Public health, Social welfare

## Abstract

**Background:**

Episodic volunteers are a critical resource for public health non-profit activities but are poorly understood. A systematic review was conducted to describe the empirical evidence about episodic volunteering (EV) in the public health sector and more broadly. Study location, focus and temporal trends of EV research were also examined.

**Methods:**

Twelve key bibliographic databases (1990-April week 2, 2014) were searched, including Google Scholar. Empirical studies published in English in peer-reviewed journals that identified participants as EVs who volunteered to support Not-for-Profit organisations in the health and social welfare sectors were included. EV definitions, characteristics, economic costs, antecedents and outcomes and theoretical approaches were examined.

**Results:**

41 articles met initial review criteria and 20 were specific to the health or social welfare sectors. EV definitions were based on one or more of three dimensions of duration, frequency, and task. EVs were predominantly female, middle aged, Caucasian (North American) and college/university educated. Fundraising was the most common EV activity and 72% had volunteered at least once. No studies examined the economic costs of EV. There was little consistency in EV antecedents and outcomes, except motives which primarily related to helping others, forming social connections, and self-psychological or physical enhancement. Most studies were atheoretical. Three authors proposed new theoretical frameworks.

**Conclusions:**

Research is required to underpin the development of an agreed consensus definition of EV. Moreover, an EV evidence-base including salient theories and measures is needed to develop EV engagement and retention strategies for the health and social welfare sectors.

## Background

Volunteering is a global phenomenon in which individuals give freely of their time, without coercion or remuneration, to a formally structured organisation with the purpose of benefitting others [1, 2]. Occurrence is widespread with rates of volunteering by continent for the period 2008–2012 averaging 37.9% in Oceania (Australia and New Zealand); 22.8% in the Americas; 19.7% in Asia; 17.2% in Europe; and 17.0% in Africa [3]. Volunteer participation on an ongoing and regular basis is vital to sustain the activities of community and non-profit organisations (NPOs) [4]. For example, in the USA, 83.9 million volunteers contributed 15.5 billion hours in the year 2000, equating to USD$239.2 billion in employee wages [5]. Of the 5.2 million Australians who volunteered in 2006, 84% contributed 623 million hours to the Australian non-profit sector with a wage equivalent value of AUD$15 billion [6]. However, volunteers increasingly face time constraints which limit their participation in traditional forms of volunteering [7, 8] and this is evidenced by declining average or median number of hours volunteered [9, 10]. Thus, the nature of volunteering is changing with preferences for more flexible, short-term, once-off, or ‘episodic’ volunteering opportunities [11].

Problematically, episodic volunteering (EV) reduces volunteer availability and increases turnover and costs for NPOs, many of whom do not have established programs or capacity to support episodic volunteers (EVs) [12]. Parallel to this, EVs are crucial when large numbers of volunteers are needed over a short-time period such as during crises [13], serving specific patient or community groups [14] or community events [15]. For example, EVs may assist in meal preparation for patients and their families in away-from-home accommodation [14] or provide care activities for marginalised groups (e.g. people experiencing homelessness [16]). As well, NPOs mobilise thousands of EVs to participate in community events (e.g. Relay For Life [17]) to raise funds for research, prevention, education and support services.

Although EV is a critical and growing social resource, research on EV is scant [15, 18–20]. Commentaries [8, 21, 22] and a descriptive review [13] on EV have identified three key aspects that are needed for research to progress in this field. First, there is no universally consistent conceptualisation of EV and definitions vary in research and practice [23]. For example, Macduff [11] proposed three classifications of EV as temporary (volunteering over a short period of hours or days); interim (regular volunteering over a short period ≤ 6 months); and occasional (returning on a consistent basis such as annually to volunteer for a short period of hours or days). By contrast, Cnaan and Handy [13] propose a continuum with volunteers participating episodically (once-off/occasionally) at one end of the spectrum and traditional volunteers at the other. Other researchers suggest a dichotomy in which once-off (ad-hoc)/short-term volunteers are compared to volunteers who participate on a regular, ongoing basis [24, 25]. In addition, definitions of EV vary further when the specific volunteering context and activity are considered. For instance, in tourism and sport contexts, event volunteers are often viewed as synonymous with EVs [26, 27], a distinction relevant for public health contexts where event volunteers provide a key source of funding for programs [28].

Second, empirical data on EV is lacking with EV prevalence and characteristics poorly described. Most general public surveys do not include specific questions on EV participation within formal organisations and likely underestimate its’ prevalence. Based on reports of the number of hours volunteered annually, it is estimated that 31% [23] to 61% [25] volunteer episodically in the USA and this compares to estimates of 38% [29] to 69% [25] in Australia. Similar rates of EV in Canada (50% to 59%) [25, 30] and the UK (44% to 78%) [25, 31] have also been estimated. Economic and social impacts of EV for volunteers and organisations also remain unquantified [32]. It has been suggested that given their infrequent participation, EVs incur less costs than volunteers who participate on an ongoing basis [13]. However, such claims rarely provide supporting evidence; consider economic costs in parallel with the social costs of EV; or examine costs to the organisation. As well, characteristics of volunteers who participate episodically have not been explored systematically [13]. Consequently, it is unclear whether there are variations in demographic characteristics within the EV population; when contrasted with longer-term volunteers; or when compared to people engaging in other forms of civic participation. In addition, potential differences in EV characteristics across sectors or activities are also unknown.

Third, EV research has seldom used theoretically driven approaches and the motivations and behaviours of EVs are not well understood [14, 15, 18, 19, 23, 33]. Drawing from broader volunteering research [4, 19, 34–37] and social-cognitive theories [38–40], key contributors to EV may include motives, attitudes, norms or social support, self-efficacy, intention, satisfaction, commitment, and self or role identity. Understanding EV behaviour and the factors contributing to EV will help to clarify patterns of EV participation over time; identify aspects on which to intervene to increase retention and the potential for transitions to more ongoing volunteering roles [13, 21]; and establish the relationship between EV and other forms of volunteering and civic participation [21].

Hence, research about EV is a key priority to inform public health more broadly and NPOs specifically who provide health and social welfare services to the community. Problematically, much of the commentary as well as the sole descriptive review on this issue [13] occurred almost a decade ago. Moreover, to our knowledge there are no systematic reviews of EV in public health or other contexts. Accordingly, we conducted a systematic review to describe the available empirical evidence for EV broadly and specifically for public health NPOs or community organisations, and from this provide recommendations for future research.

As the first systematic review on EV, we initially examine the current state of EV research by describing location, focus and temporal trends of published EV research. For the purposes of building an evidence base and in line with our first aim to describe EV conceptualisations and characteristics, we then address definition and empirical aspects for all EV articles initially assessed for eligibility, regardless of the sector in which these studies occurred (i.e. broad review). Consistent with our second aim to understand EV behaviour and contributors to EV in a public health context, we then narrow our review to focus only on those articles describing EV in the health and social welfare sector and address the antecedents, outcomes and theoretical aspects (i.e. focused review).

## Methods

In line with these aims, key questions were developed by two authors to guide the broader and focused review, and finalised after consultation with a third author. Questions were as follows:

1. Definition
  How was EV conceptualised or defined?
2. Empirical
  What was the prevalence of EV?
  What was the economic and social value of EV for volunteers and/or NPOs?
  What were the demographic characteristics and volunteering activities of EVs and in which sector did they participate?
  What EV antecedents and outcomes were examined? (health and social welfare sector only)
3. Theoretical
  What volunteering theories were used to understand EV? (health and social welfare sector only)

The review and subsequent reporting of results were guided by the PRISMA statement [41]. Ethical approval was not required.

### Search strategy

Medline and PsycINFO (via Ovid) databases were searched initially (1990– Week 2, April, 2014). Search keywords were derived from terminology commonly used in volunteering articles to describe episodic volunteering and included the following: ((episodic.mp OR sporadic.mp OR occasional.mp OR short term.mp OR irregular.mp OR ad hoc.mp) AND (volunteer$.mp) AND (volunteer$.ab OR voluntar$.ab)). A second more focussed search was conducted on EbscoHOST, ProQuest, Science Direct, Web of Science, Wiley, Ingenta Connect, Taylor & Francis, JSTOR, and SAGE databases with the term: “episodic volunteer*”. Duplicates were removed prior to examination of article titles and abstracts. A further focussed search with the term “episodic volunteer*” and cited reference searches were conducted on Google Scholar to supplement the electronic database searches [42]. Reference lists of relevant retrieved articles were also searched by hand.

Potentially relevant articles were identified by examining the title and abstract and then retrieved for more detailed evaluation against the inclusion criteria by two authors. Only peer-reviewed journal articles describing empirical studies were included. Any variation in inclusion/exclusion decisions was discussed until consensus was reached. Qualitative and quantitative studies were included if they met the following pre-determined criteria: empirical studies that identified the whole sample or a sub-sample as EVs (i.e. short-term, set timeframe, often project or event-based, occurring one time only or repeated on an annual or seasonal basis) [11, 13, 15, 33]; AND EV occurred within a person’s own country of residence outside of an emergency/disaster situation; AND EV was for the purpose of supporting NPOs or community organisations providing health and social welfare services to the community; AND published in English after 1st January 1990 and prior to April Week 2, 2014. Empirical studies that included EV for purposes other than that described above were noted but did not undergo quality assessment or full review.

The methodological quality of studies included in the review was assessed independently by two authors using separate criteria for qualitative and quantitative research. Differences were resolved by consensus. Qualitative studies were assessed using criteria in the literature deemed to denote high quality [43–45] and included: sampling frame described, justified, or met; framework for study design, methodology and orientation disclosed; interviewer bias addressed; method of analysis described; inclusion of reliability and validity checks; clear presentation of data. Quantitative cross-sectional and prospective studies were assessed using criteria adapted from established tools for cohort and case–control studies [46] and included: representativeness of the study sample (participant selection), measures applied (reliability and validity), attrition bias (participation rates modified to suit the context of mail and online surveys with average response rates of 35% [47] to 65% [48], respectively), and evidence of follow up. Characteristics and results of included qualitative and quantitative studies were summarised in tables by one author and verified independently by a second author.

## Results

### Search results

**Figure 1:**
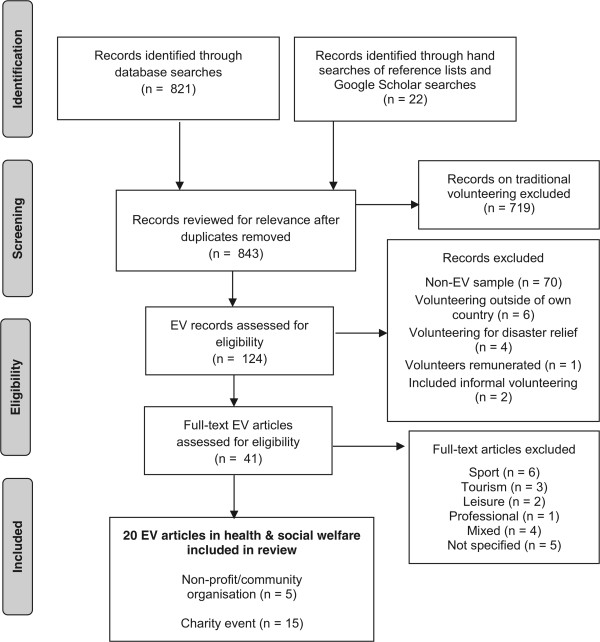
**PRISMA flow diagram of systematic review inclusion and exclusion process.**

The process of identifying articles for the review is outlined in Figure 1. The database searches, Google Scholar searches, and reference lists searched by hand identified 843 articles. On examination of titles and abstracts, 124 were potentially relevant and after checking against inclusion criteria 41(reporting on 43 studies) were retrieved for further evaluation (i.e. broad review). After 23 articles were excluded which either did not include separate data from EVs in health and social welfare or did not specify the organisation/sector volunteered for, a total of 20 articles (reporting on 22 studies) met all inclusion criteria and were retained for the focused review: 16 were cross-sectional descriptive quantitative studies; 5 were cross-sectional descriptive qualitative studies; and 1 included both cross-sectional and prospective descriptive quantitative studies.

### Location, study focus, and temporal trends

In describing available EV research, we examined location (country) and temporal (year published) trends according to study focus. Study focus comprised all empirical articles on EV which met initial criteria (i.e. regardless of sector; n = 41); empirical articles on EV in the health and social welfare sector specifically (n = 20); and within this sector, articles which focused on EV for an NPO/community organisation program (n = 5) or charity sport event (n = 15). EV studies overall were conducted primarily in North America (USA n = 20, 48.8%; Canada n = 5, 12.2%), and this trend was also consistent for the health and social welfare sector (USA n = 16; Canada n = 3). EV studies were also conducted in Australia (n = 7, 17.2%), European countries (Switzerland n = 2; Norway n = 1; Belgium n = 1; France n = 1; UK n = 1; 14.6%), and 3 were from multiple countries; however, of these only two European studies focussed specifically on the health and social welfare sector (Belgium n = 1; France n = 1).

All except two EV articles were published since 2002, with a comparison over the specific years 1999–2003 (n = 2) and 2009–2013 (n = 22) showing approximately a tenfold increase in the number of studies. A sixfold increase in studies over time for the health and social welfare sector is also evident (1999–2003, n = 2 vs. 2009–2013, n = 12). However, this increase can be attributed to the greater number of studies on charity sport events in this sector. By comparison, the number of studies focussing on other EV activities such as volunteering for a program hosted by an NPO/community organisation (e.g. homeless shelter) did not increase.

### Definition

#### EV Attributes

Of 41 articles matching initial criteria, 20 did not provide a definition of EV. However, 19 of these studies included event volunteers who by their nature of participation are considered by some researchers as synonymous with EVs (e.g. [26, 27]). In the remaining articles [14–16, 18, 24–27, 32, 49–60] EV was described according to one or more of the following dimensions: duration of participation (e.g. short-term [60]); frequency of participation (e.g. 1 or 2 occasions [15]); and task (e.g. project-based [59]). EV definitions based on duration and frequency were the most common (n = 6, 28.6%); followed by a definition including all three dimensions of duration, frequency and task (n = 4, 19%); duration and task (n = 3, 14.3%); frequency only (n = 3, 14.3%); duration only (n = 2, 9.5%); task only (n = 2, 9.5%); and frequency and task (n = 1, 4.8%). For the health and welfare sector only, 2 articles defined EV using the dimensions of duration, frequency and task; 2 used frequency only; 1 duration and frequency; and 1 task only.

### Empirical

#### EV prevalence and participation patterns

Of the 41 studies matching criteria regardless of sector, 29 recruited a sample comprised entirely of EVs [15, 16, 26, 55–57, 59–79]; 8 included a sub-sample of EVs, the proportion of which ranged from 19% to 68% [14, 15, 18, 24, 25, 27, 58, 80]. Six did not specify the proportion of EVs included [32, 49–51, 53, 54]. In 24 studies, participation patterns of EVs based on frequency of volunteering ranged from 0 (i.e. first time) to more than 12 times. There was no consistency across studies in the use of frequency of volunteering to classify or describe EVs, with studies most often considering all EVs as one homogenous group. However, the majority of studies included EVs who had participated at least once (72%) rather than EVs participating for the first time (28%). Specifically, 9 studies included first-time EVs [18, 58, 63, 65, 66, 71–73]; 7 included EVs who had participated 1 or 2 times [15, 18, 64, 65, 70, 75]; 6 included EVs participating 3 or 4 times [14–16, 18, 64, 77]; 3 included EVs who had participated more than 5 times [16, 18, 55]; and 7 reported prior EV but did not specify the number of times volunteered [26, 56, 58, 70, 71, 73, 74].

#### Economic and social value of EV

No studies were identified that reported the value or costs of EV for individual, NPOs/community organisations, or the sector. However, one study in the health and social welfare sector [32] examined the importance of the potential benefits of volunteering for EVs. Appreciation by staff and families was an important benefit for EVs (45.7%), followed by free parking (22.7%). By contrast, a much smaller proportion rated the benefit of recognition in either a public (4.5%) or private (e.g. thank you note; 17.9%) forum as important. Although increasing social ties was a benefit for ongoing volunteers, less than half of EVs formed close social connections with other volunteers (44.6%). Volunteering was rated as more important than leisure and work by 39.7% and 22.1% of EVs, respectively. However, few (7.4%) rated EV as more important than friends or family. As well, EVs were asked to estimate the monetary value of their contribution to the organisation. Compared to ongoing volunteers (US$12.06 per hour), EVs underestimated the value of their contribution (US$8.10 per hour) and this difference was statistically significant [32]. In one article on charity sport event participation [75], EVs agreed or strongly agreed that their future participation would be constrained by: not having an opportunity to participate near home (18.2%); difficulty finding others to participate with (17.4%), and not being asked to participate in an event (15.8%).

#### EV sample characteristics

Of the 41 articles (reporting 43 studies) identified, 12 did not report demographic characteristics for EVs. Of the remaining studies, 16 provided an average age for their EV sample and this ranged from 20.4 years to 45.0 years; 14 provided an age range (most commonly 30 to 60 years); and 2 did not specify age. 28 studies reported gender, with the proportion of males ranging from 10% to 100%; and 32.8% to 100% for females. Less than half of included studies reported ethnicity, with the remainder reporting predominantly Caucasian samples (49.99% to 100%). In 10 of 14 studies reporting level of education, ≥ 65% of EVs had completed or were undertaking a university/college degree. Few studies reported relationship status (n = 8); employment (n = 10); or income (n = 8). Of these, the majority in each EV sample were married (range 36% to 79.8%); employed full or part-time (range 57% to 94%); and represented at least middle income levels (USD ≥ $50,000).

#### EV sector and activities

Thirty-two articles identified the sector in which EV occurred and the specific activity (or activities) undertaken by EVs. Sectors represented included: sport; tourism; leisure; professional; and health and social welfare (as detailed in Figure 1). In 23 articles (71.8%), EV involved event volunteering with tasks including fundraising (n = 14; 68.9%) or operations (e.g. event set up/pack up; ticket sales; transport) (n = 9; 39.1%). Fundraising activities occurred mainly in the health and social welfare sector and operations activities were reported only in the sport and tourism sectors. The remaining articles comprised social service/client care (e.g. counselling, meal preparation) (n = 5; 15.6%) or instructional (e.g. training, tourist guide) (n = 2; 6.3%) activities and short-term community projects (e.g. building renovation) (n = 2; 6.3%).

#### EV antecedents and outcomes (focused review)

Table 1 displays the main findings for studies which met final criteria for the focused review. The lack of consistency in predictor and outcome variables prevented us from drawing conclusions regarding similarities in key findings across studies. However, motives for EV were the most frequently examined antecedent and outcome variable (n = 10), thus we focused on describing the underlying motivational structure of EV. The most common motives for EV included: 1) supporting the cause, helping others, or civic duty/obligation; 2) psychological (e.g. self-esteem) or physical (e.g. physical challenge) enhancement, goal accomplishment as an individual or group; and 3) socialising, enjoyment, building connections with others. Other EV motives that were less common included developing knowledge, skills or competency; and having a personal connection to the cause (e.g. illness), or giving back. Helping others and social motives were also related to frequency of EV [59], organisational-based self-esteem [78], satisfaction with EV and intention to volunteer episodically in the future [77].

**Table 1:** **Summary of articles included in the focused systematic review of EV in the health and social welfare sector**

Study details	Sample*	Volunteering theory	Independent variables	Outcome/s	Main findings
***Quantitative studies***					
Allison [59] 2002 (USA)	N = 195	None	Method of assessment of motivations (Volunteer Functions Inventory vs. open-ended probe).	Frequency of volunteering for MAD	• VFI: The most salient motive was values (M = 6.10), followed by understanding (M = 4.76) and esteem (M = 4.37). Average scores on the remaining motives were below the scale mid-point.
	22% male.				
Quantitative cross-sectional	89% Caucasian.				
	Served at some point in last 8 yrs.				
					• Open-ended probe: coded responses most often reflected the esteem motive, followed by the value motive.
	Make A Difference				
					• VFI motives but not motives identified by the open-ended probe measure predicted frequency of volunteering for MAD (R^2^ = .13)
					• Increased VFI value scores (β = .23, *p* < .05) and decreased VFI social scores (β = -.19, *p* < .05) significantly predicted an increase in frequency of volunteering for MAD.
Beder [60] 2008 (USA)	N = 633	None	Event group (four events for different causes and involving varying levels of volunteering)	Motivation (Volunteer Motivation Inventory)	• The five most highly rated motives overall were values; self-esteem; understanding; reactivity; and protective.
	Police crisis fund; young amputees; ovarian cancer; breast cancer.				
Quantitative cross-sectional					• Participants in the charity event for breast cancer scored higher on the values (expression/action for beliefs of the importance of helping others); interaction (building social networks and enjoyment of interaction with others); and physical (physical challenge and endurance) motives subscales.
					• 54.8% overall stated they volunteered for the event because of the cause it represented; and within this 79.8% indicated they volunteered for the breast cancer charity sport event because of the cause.
Filo [70] 2011 (USA)	Study 1 N = 568	None	Recreation and charitable giving motives.	Attachment to the event.	• Study 1: social, reciprocity, self-esteem, need to help others, and desire to improve the charity motives predicted event attachment (Adj R^2^ = .47).
	46.3% 40-64 yrs.				
Quantitative cross-sectional	74.6% Caucasian.				
			Prominence of charitable cause in marketing of event (high vs. low prominence).		• Study 2: Intellectual, social, physical, escape, reciprocity, self-esteem and desire to improve charity motives predicted event attachment (Adj R^2^ = .35).
	Lance Armstrong Foundation;				
	Study 2 N = 689				• Stronger contribution of charitable motives for event with more prominent charitable cause vs. stronger contribution of recreation motives for event with less prominent charitable cause.
	34% male.				
	70% 25-44 yrs.				
	The Capital Area Food Bank of Texas				
Filo [74] 2012 (USA)	N = 568	None	Motives for participating in the event (social, physical, escape, charity); Belief in making a difference	Attachment to the event.	• Belief in making a difference partially mediated the effect of social and charity motives on attachment.
	18-70 yrs.				
Quantitative cross-sectional	74.6% Caucasian.				
	Lance Armstrong Foundation			Belief in making a difference.	• Significant paths were present from social motives and charity motives to belief in making a difference.
Harrison [16] 1995 (USA)	N = 157	Author proposed Theory of Episodic Volunteer Motivation	Intention to attend volunteer work at the shelter; Intention to stay home; Intention to socialize or recreate.	Volunteer attendance.	• Intention to attend volunteer work was a significant and consistent predictor of attendance in all samples.
	All male.				
Quantitative cross-sectional and prospective	Served at least 2 nights previously.				• Intentions to attend competing alternatives (home, social/recreate) were predictors of volunteer attendance in the cross-sectional study samples (predicting past volunteer work) but not the prospective study sample.
	Homeless shelter				
					• Experience volunteering appeared to moderate the impact of competing alternatives on attendance.
			Attitude; subjective norm; perceived behavioural control; moral obligation.	Intention to attend volunteer work at the shelter.	• The impact of attitude, subjective norm, perceived behavioural control and moral obligation on attendance was mediated via intention to attend
					• In both the cross-sectional and prospective study samples, subjective norm, perceived behavioural control, and moral obligation were significant predictors of intention to attend volunteer work. Attitude was a significant predictor of intention to attend volunteer work in the cross-sectional study samples only.
					• Experience appeared to moderate intention to attend volunteer work such that the more experienced a volunteer became, the less anticipated satisfaction from volunteer work impacted on their motivation to volunteer.
Haski-Leventhal [32] 2011 (USA)	N = 258	None	Volunteer type (ongoing vs. episodic)	Satisfaction with volunteering; benefits; relationships; relative importance; charitable giving.	• EV: 92.8% satisfied with volunteering tasks; 95.7% satisfied with appreciation from families; 94.4% satisfied with appreciation from staff; 88.2% satisfied with their relationships with other volunteers; 59.3% satisfied with their training; 73.3% satisfied with flexibility of volunteering.
	No EV specific demographics.				
Quantitative cross-sectional	Philadelphia Ronald McDonald House				
					• EV: Appreciation by staff and families (45.7%), free parking (22.7%), and a thank you letter (17.9%) were most important benefits.
					• EV: 44.6% formed close relationships with other volunteers.
					• EVs donated money to organisation (22.5%) or gave other forms of in-kind support (49.3%).
					• Volunteering rated as more important than work (22.1%), leisure (39.7%), and friends/family (7.4%) by some EVs.
					• EV’s valued their contribution at US$8.10 per hour (a statistically significant difference to ongoing volunteers who rated their contribution at US$12.06 per hour).
Hustinx [18] 2005 (Belgium)	N = 652	Author proposed analytic framework to identify Styles of Volunteering.	Structural (e.g. length of service, intensity of involvement) and cultural (e.g. identification with organisation) indicators of volunteering.	Style of volunteering	• Five different styles of volunteering: episodic contributors, established administrators, reliable co-workers, service-oriented core volunteers, and critical key figures.
	No EV specific demographics.				
Quantitative cross-sectional	Red Cross				• 139 (21%) classified as episodic contributors.
					• Episodic contributors characterised by: infrequent volunteering (once or several times a year); low number of monthly hours (≤ 4 hours per month); do not perform core activities (e.g. board membership); perform one activity; and identify weakly with the organisation or volunteering.
					• Most episodic contributors had been involved ≥ 2 years (1/3 for ≥ 5 years).
Hustinx [14] 2008 (USA)	N = 258	Author proposed net cost theory.	Type of volunteer (regular vs. episodic)	Demographic characteristics; years of volunteering; type of activity; motivations; satisfaction with volunteering; importance of rewards	• Compared to regular volunteers, episodic volunteers were more likely to be: younger (M_age_ = 40.8 yrs); employed full-time; volunteered for less years on average (2.9 yrs); participate in the guest chef program (84%).
	M_age_ = 40.8 yrs.				
Quantitative cross-sectional	32.3% were episodic volunteers.				
					• Compared to regular volunteers, episodic volunteers more frequently: emphasised social motives (e.g., someone asked them to volunteer; friends/family volunteer); felt driven by a civic or religious sense of duty; viewed their contribution as a way to make their community a good place to live; emphasise value-based motives as driving their participation.
	Ronald McDonald House Charities				
					• Regular and episodic volunteers expressed similar levels of satisfaction overall.
					• Regular volunteers placed more importance on rewards than episodic volunteers.
					• Appreciation by staff and family was the most important reward for both types of volunteers.
Mayer [78] 2007 (USA)	N = 93 team leaders	None	Organisational-based self-esteem; Frequency and length of participation.	Motivation (Volunteer Functions Inventory); Organisational-based self-esteem;	• Values, social, understanding and sense of worth motives had highest mean ratings.
Quantitative cross-sectional	28% male.				• Understanding, sense of worth, social, and values (but not career) motives were significantly related to organisational-based self-esteem.
	72% 21-50 yrs.				
	91% Caucasian				
	American Cancer Society				• People who volunteered more often (>10 days per yr), and for a longer time (> 10 yrs) had higher organisational-based self-esteem scores than those who volunteered for <10 days per yr and for <10 yrs.
					• There was no difference on organisational-based self-esteem scores as a function of intention to continue volunteering for more or less than 15 yrs.
Rundio [80] 2014 (USA)	N = 170	None	Event type (Cause vs. non-cause-related).	Motives (revised version of Motivations of Marathoners scale).	• Most important motives for participation in cause-related events were: personal goal achievement, general health orientation, self-esteem, weight concern, and affiliation with others.
	M_age_ = 37.16 yrs.				
Quantitative cross-sectional	43.5% male.				
	Cancer; Big Brothers, Big Sisters				
Snelgrove [67] 2010 (USA)	N = 206	None	Motives for participation.	Experience (first-time vs. repeat participants).	• For first time participants, strongest motivators were physicality, a desire to support others, and socializing.
	M_age_ = 41.3 yrs.				
Quantitative cross-sectional	61% male.				• For repeat participants, strongest motivators were supporting others, cycling identity, and physicality.
	MS society				
					• Repeat participants compared to first-time participants had a significantly stronger MS fundraiser and cycling identity; and a significantly lower physicality motive.
Won [77] 2010 (USA)	N = 211	None	Motives for participation;	Satisfaction with the event; future intention to participate in the event.	• Motives for participating in charity sport events were represented by a six-factor solution that explained 66.2% of the variance.
	41% male.				
Quantitative cross-sectional	M_age_ = 35 yrs.		Gender; Age.		
	92.9% Caucasian.				• These motives were: Philanthropy (altruistic motivations, helping the cause or organisation); Social/Entertainment (social needs, enjoyment); External/Benefits (future benefits from the event); Family needs (satisfying family needs); Sports (enjoyment of sport activities); and Group collaboration (working together as a group).
	Participated in event for 2.83 yrs on average.				
	American Cancer Society				
					• Philanthropy was the most important motive followed by Family needs, Group Collaboration, and Social/Entertainment.
					• The motives explained 44% of the variance in satisfaction with the event and 15% of the variance in intention to participate in future.
					• Increased Philanthropy (β = .58) and reduced External/Benefits (β = -.14) motives significantly predicted Satisfaction with the event.
					• Increased Philanthropy (β = .35) and increased Family needs (β = .15) motives were significant predictors of intention to return in future.
					• Philanthropy was a significantly more important motive for females than males.
					• External/Benefits was a significantly more important motive for males than females.
					• Younger participants (especially younger males), compared to older participants viewed Social/Entertainment as a more important motive.
Won [75] 2011 (USA)	N = 247	None	Participation type (voluntary – own choice vs. non-voluntary – asked by someone else to participate)	Information source for volunteering and charity-related information; motivation to participate; constraints	• 66.1% stated friends/relatives as the primary source of event information.
	20.6% male.				
Quantitative cross-sectional	Mage = 37.0 yrs.				• Based on confirmatory factor analysis of the underlying motivational structure, the key motivations for participation were: supporting the MS society, socialisation, and sport.
	70.9% Caucasian.				
	Multiple Sclerosis Society				
					• Based on confirmatory factor analysis of constraints showed that external constraints (access, cost, social isolation) served as greater barriers to participation than internal constraints (lack of interest, time or energy).
					• Compared to voluntary participants, non-voluntary participants (asked by someone else) were more motivated by social aspects of the event, and were more likely to return to an event in future if they are asked to participate.
					• Voluntary participants were more likely than non-voluntary participants to return to an event of their own free will and were more likely to donate in future.
Wood [69] 2010 (Canada)	N = 206	None	Self-identity; social identity; demographics; location; type of involvement (team or individual)	Past event participation; Past amount fundraised.	• Four segments of volunteers were identified: event enthusiasts (cause and sport identity; 31%); cause fundraisers (cause only identity; 13%); road warriors (sport only identity; 36%); and non-identifiers (20%).
	62.6% male.				
	Multiple Sclerosis Society of Canada				
Quantitative cross-sectional					• The event enthusiasts segment raised more money on average and differed significantly from road warriors and non-identifiers (but not cause fundraisers).
					• Event enthusiasts (M = 7.17 events) reported significantly greater past event participation than all other segments.
***Qualitative studies***					
Filo [72] 2008 (USA)	N = 31	None	None	Attraction to the charity sport event; charitable giving; motives	• Motives (intellectual, social, competency, reciprocity, self-esteem, need to help others, and desire to improve the charity) contribute to attraction to the event.
	61.3% male.				
Qualitative cross-sectional	100% Caucasian.				
	Lance Armstrong Foundation				• The charitable aspect of the event informed social and competency motives and strengthened the connection felt to the event.
Filo [73] 2009 (USA)	N = 35	None	None	Participant attachment to the event	• Three themes emerged that were proposed to inform attachment to the event: *camaraderie* (e.g., being part of something bigger for a common cause, belonging, solidarity, surrounded by like-minded others); *cause* (making a difference by raising awareness and supporting a worthy cause, inspiring and being inspired by others); and *competency* (health and fitness, physical challenge, enjoyment).
	50% male.				
Qualitative cross-sectional	96.9% Caucasian.				
	Lance Armstrong Foundation				
Filo [71] 2013 (USA)	N = 46	None	None	Brint’s (2001) typology of Gemeinschaft-like structural and cultural properties of community	• Five of the six properties of community were present: dense and demanding social ties; social attachments to and involvement in institutions; ritual occasions; perceptions of similarity with others; and common beliefs in an ideas system, moral order, institution or group.
	40-64 yrs.				
Qualitative cross-sectional	89% Caucasian.				
	Lance Armstrong Foundation				
Scott [66] 2003 (USA)	N =11	None	None	Motivation for participation and experience at event.	• Motives for participation in order of most salient: personal connection to the illness; social benefits; supporting the cause/community obligation; fitness; fundraising.
	30-50 yrs.				
	27.3% male.				
					• Experience at event (what they saw, how it made them feel): participants commented on survivors, pink shirts, bald women, number of people attending; and corporate sponsorship and support. Participants consistently reported mixed emotions.
Qualitative cross-sectional	91% Caucasian.				
	Susan G. Komen Breast Cancer Foundation				
Snelgrove [68] 2013 (Canada)	N = 57	None	None	Formation of attachment to the event	• Participants developed attachment to the event in three ways: 1) being known as a fundraiser (e.g. public recognition, close others and society aware they were doing good for the organisation); 2) aligning self and cause (e.g. increased their comfort telling others and talking about their disease); and 3) developing social bonds (e.g. feeling part of a larger group working toward a common goal of ending MS; walking for loved ones initially but then over time this extended to people they met at the walk and people with MS who they did not know).
	10% male.				
	18-57 yrs.				
Qualitative cross-sectional					
	Involved in event for ≥ 5 years.				
	Multiple				
	Sclerosis (MS) Society of Canada				
***Quantitative and Qualitative***					
Hendriks [65] 2013 (Netherlands)	N = 189	None	Motivation for participating	Personas	• Six factors explained 62.4% of variance in motivations. These were: well-being (e.g. enjoy the sport and a healthy lifestyle); humanity (e.g. support those affected by cancer, participate to remember a loved one); social (e.g. to be with friends, to increase self-image or social worth); cause (e.g. support the cause or the organisation); empowerment (e.g. make cancer a national priority); and personal (e.g. personally affected by cancer/survivor).
	67.2% male.				
	M_age_ = 42.02 yrs.				
Quantitative and qualitative cross-sectional	74.1% first time participants.				
	Alpe d’HuZes event (Cancer)				
					• Four personas created based on clustering of motivations: health junkies (motivated by well-being factor, 20%); promoters (motivated by cause and empowerment factors, 28.8%); legends (motivated by personal factor, 29.6%); and caretakers (motivated by social factor, 21.6%).

*Details included where specified.

### Theoretical (focused review)

Novel volunteering frameworks were proposed by authors of three articles included in the focused review: theory of episodic volunteer motivation [16]; net cost theory [14]; and styles of volunteering [18]. The theory of episodic volunteer motivation is an extension of the theory of planned behaviour (a decision-making model in which intentions are proposed to predict behaviour; intentions are predicted by attitude, subjective norm, and perceived behavioural control). In extending this latter model, the theory of episodic volunteer motivation incorporates intentions to participate in competing activities other than volunteering (e.g. staying home, leisure), and perceived moral obligation to volunteer (i.e. volunteering as the ‘right’ thing to do). Also, a feedback loop is proposed which suggests that the predictors of EV participation change over time [16]. Net-cost theory proposes that although volunteers are not paid for their contribution they incur costs as a result of volunteering. These costs are proposed to vary based on intensity of participation, activity undertaken and benefits associated with volunteering. Regular volunteers are expected to experience greater costs than EVs, and volunteering motives and perceived benefits are expected to vary between these two groups [14].

Styles of volunteering is an analytic framework which draws on structural (e.g. length of service) and cultural (e.g. identification with or commitment to organisation) indicators of volunteering to propose five styles of volunteering, of which one is episodic contributors [18]. Episodic contributors are characterised by their infrequent volunteering (once or several times a year); low number of monthly hours (≤4 hours per month); performance of a single peripheral activity (e.g. does not have board membership); and weak identification with the organisation or volunteering. Although other studies did not use a volunteering framework, they applied approaches from other disciplines including the Psychological Continuum Model [70, 72–74]; identity theory [67, 69]; and functional/symbolic theories of motivation [59].

### Study quality

#### Quantitative studies

All quantitative studies were classified as Level IV evidence studies [81]. Given the nature of the samples all were selected or highly selected groups limiting generalizability of findings beyond the context in which episodic volunteers were studied. With this in mind however, 60% used samples of participants who were representative of the larger EV sample [18, 59, 65, 67, 69, 70, 74, 75, 80]. Ten studies used previously validated and reliable tools [18, 59, 60, 67, 69, 70, 74, 75, 78, 80]. The remainder used novel measures and provided validation or reliability data [14, 16, 32, 65, 77]. Participation rates ranged from 8.4% to 100%. Five studies reported high participation rates (i.e. >65% [48, 82]) [16, 18, 67, 69, 78] with response rates for the remainder of studies either average (i.e. 35% to 65% [48, 74]) (n = 1 [80]), low (i.e. <35%) (n = 7 [14, 59, 60, 65, 70, 74, 75]) or not described (n = 2 [32, 77]). The single prospective study included 100% of the initial sample at follow-up [16].

#### Qualitative studies

All qualitative studies were Level III evidence descriptive studies [37]. All except one qualitative study [66] provided a clear rationale for sample selection and all studies adequately or partially described the sample. Three of the five qualitative studies provided and met a rationale for sample size [71–73]. One study described a qualitative framework and addressed interviewer bias [66]. All studies used an objective method for data capture (audio-recording), provided some level of description about the method of analysis used, and all except one study included checks for data credibility. All studies provided example quotes to aid understanding of data interpretation and analysis.

## Discussion

Although EV is vital for the public health sector there are critical gaps in the research to date that limit our understanding and knowledge. These critical gaps are: the lack of definitional clarity on EV; the use of atheoretical approaches; and the absence of an assessment of EV economic/social value or costs. First, only half of the studies reviewed defined EV, with duration and frequency the most common dimensions used. This discrepancy may be explained by difficulties conceptualising EV [13] or the focus on event volunteering in the majority of EV research which involves activities that are clearly delineated by these dimensions (e.g. fundraising). By contrast, EV involvement in health and social welfare NPO or community programs may be less clear cut and potentially more difficult to define. In addition, Leonard and Onyx [54] note that EV roles may be less available in health and social welfare with more opportunities for EV in sport, leisure or tourism sectors. Thus, an immediate priority for future research is the development of an agreed operational EV definition derived from prior EV research and the expert opinion of EVs, researchers, NPO staff, and key informants in public health and other sectors more broadly.

Second, although three studies proposed novel theoretical frameworks, none used previously tested volunteering theories. Two traditional volunteering theories that have not been applied in EV research but may be of relevance are the Volunteer Process Model (VPM) [4] and the Three-Stage Model of Volunteer’s Duration of Service [34]. Briefly, the VPM identifies the key features of individual volunteering and structures these within three linked stages: antecedents (e.g. demographics, motives); experiences (e.g. satisfaction, organisational commitment) and consequences (e.g. intention to volunteer in future, retention) [4]. As a complement to the VPM, the Three-Stage Model stipulates when these specific antecedents and experiences may be most influential over a short (≤12 months) and long-term (>1 year) duration, and proposes intention to continue volunteering at each time-point as the main link between these variables and volunteering behaviour [34, 83, 84].

Drawing from previously tested theoretical approaches will assist in building an evidence base for EV, and enabling comparisons with forms of traditional volunteering in which EVs may also participate [15]. Thus, we propose an integration of these two models to form an Episodic Volunteer Engagement and Retention (EVER) model [17]. The EVER model, informed by broader EV research, proposes that EV antecedents, experiences, and consequences evolve over time and offers guidance as to the critical phases when these changes may occur: after the first EV experience (Novice); during a 2 to 4 year period of EV on an irregular basis (Transition); and following 5 or more years of consecutive, regular EV (Sustained). Antecedents of EV are also proposed to differ based on EV phase with motives, social norm, and satisfaction critical for Novice EVs; social norm, satisfaction, sense of community and organisational commitment important for Transition EVs; and social norm, psychological sense of community and organisational commitment important for Sustained EVs. In all phases, intention to continue EV is proposed as the most proximal link to EV. Although in the initial stages of validation [17] and likely requiring further refinement to include constructs such as self-efficacy [16, 37, 85] and identity [35, 36] which may be critical for EV retention, the EVER model may serve as a guiding framework for future research. Empirical prospective tests using the EVER model framework will enable identification of pathways to EV retention based on duration of volunteering, and may also prove efficacious as a basis to inform understanding about the potential link between EV and more traditional forms of volunteering.

Perhaps as a result of atheoretical approaches, there was little consistency across studies in the EV antecedents and outcomes examined. Motives emerged as an antecedent and outcome variable that was frequently examined. The underlying motivational structure of EV primarily reflected helping, social and personal or physical development with developing knowledge or skills and having a personal connection to the illness or reciprocity less prominent. However, approaches to the measurement of motives varied and this introduced difficulties in identifying similarities in motives across studies reviewed. The consistent use of measures, such as the Volunteer Functions Inventory [86], that are widely applied to assess the motives of ongoing volunteers [19] would facilitate comparisons of motives within and across EV sectors, and traditional volunteering studies.

Third, research on constraints and benefits associated with EV were scant, and no studies were identified in the course of this review which estimated the economic or social value and costs of EV for volunteers, NPOs, the health and social welfare sector or society more broadly. For NPOs, EV recruitment, training, management, infrastructure and insurance are likely costs, whereas time contributed by EVs is a clear benefit to the organisation [6]. For EVs, skill acquisition or training and expanding social networks are benefits which may be balanced by transport and child-care costs, specific resources needed for the EV role, and time lost for other social or family activities [87]. Adopting an EV and NPO perspective to identify EV costs and benefits in established EV programs would serve as an initial step in advancing knowledge and provide primary descriptive benchmarking data for future use in the health and social welfare NPO sector.

### Study quality and trends

Research reviewed comprised low level evidence, cross-sectional descriptive qualitative (Level III) or quantitative (Level IV) studies, with only one study combining cross-sectional and prospective research. The number of EV studies in the public health sector increased sixfold over a 10 year period. The increasing number of studies and the predominance of descriptive cross-sectional research is consistent with expectations for a field of research in the developmental phase [88]. Of concern, international EV research efforts in health and social welfare have not developed equally across the globe. Research was dominated by a North American perspective with few studies conducted in Europe and none in the UK. Some Asia-Pacific regions traditionally have a strong civil society, including high rates of volunteering and giving [3], yet this perspective was also absent from EV research in this sector. In addition, reporting of sample characteristics was not consistent. The average demographic profile of samples in studies reviewed comprised EVs who were predominantly North American, middle aged; female; Caucasian; and university/college educated; and had undertaken EV at least once in the past. Idiosyncrasies of this research have implications for generalising findings beyond the studies reviewed. Cross-cultural, demographically-diverse, prospective and experimental studies that use consistent theoretical and measurement approaches are needed to move EV research beyond this current phase.

## Conclusion

Although EV is vital for the public health sector and the evidence base on EV is growing, there are critical gaps in the research to date. These limitations include lack of definitional clarity; the near absence of theoretically driven EV research and as a consequence little consistency in antecedents and outcomes examined. Articles reviewed comprised low level evidence studies with a predominantly mono-cultural focus. Future research is required to underpin the development of an agreed consensus definition of EV. From this, prospective research is needed that applies salient volunteering theories and validated measures to build an EV evidence-base and develop EV engagement and retention strategies for the public health sector and more broadly.
